# When motivation becomes feeling: disentangling the relationship between motivational beliefs and emotions in elementary and secondary physical education

**DOI:** 10.3389/fspor.2026.1869770

**Published:** 2026-07-09

**Authors:** Michael Braksiek, Iris Pahmeier, Kelly L. Simonton

**Affiliations:** 1 Institute of Sport Science, Faculty II, University of Vechta, Vechta, Germany; 2 Division of Kinesiology & Health, College of Health Sciences, University of Wyoming, Wyoming, WY, United States

**Keywords:** classroom climate, control-value theory, need-supportive teaching, self-determination theory, track field

## Abstract

**Purpose:**

Affective learning in physical education (PE) depends not only on whether students feel good or bad, but on how specific emotions develop in relation to motivational experiences and teaching conditions. This short-term longitudinal study examined whether autonomous and controlled motivation predicted changes in discrete emotions during PE and whether these associations differed between elementary and secondary classes.

**Method:**

Students from German elementary and secondary schools completed questionnaires before and after approximately five-week track-and-field units (*N* = 375; 20 classes). Autoregressive mixed-effects models predicted T2 enjoyment, boredom, anger, and shame from T1 motivational regulations while controlling for the corresponding T1 emotion, school level, gender, and class-level need-supportive teaching. Reverse models tested whether T1 emotions predicted changes in motivation.

**Results:**

Autonomous regulation predicted higher T2 enjoyment (*b* = 0.21, *p* = 0.003) and lower boredom (*b* = −0.13, *p* *=* 0*.*035). Need-supportive climate related to higher enjoyment and lower boredom and shame. Controlled regulation showed no unique associations. The association between autonomous motivation and enjoyment was stronger in secondary than elementary classes (interaction b = 0.31, *p* = 0.005). Reverse models showed no evidence that T1 emotions predicted changes in motivation.

**Discussion/conclusion:**

Across short PE units, autonomous reasons for participation and need-supportive teaching appear to be linked to more favorable emotional trajectories. These findings support an affective-learning perspective in which students’ emotions are not merely outcomes of PE participation, but developmentally sensitive indicators of how motivational meaning is formed in instructional contexts.

## 1 Introduction

Physical education (PE) is a powerful context for shaping how young people feel about movement. Moments that are enjoyable in class foster engagement, learning, and the likelihood of being active beyond school; whereas boredom, anger, or shame can undermine participation and leave lasting negative impressions in different ways [e.g., (1)]. Discrete emotions experienced in PE can vary greatly by valence (i.e., positive and negative), magnitude (i.e., associated level of arousal), and by focus (i.e., emotions associated with learning process or outcome of learning). Emotions are also responsive to how lessons are taught and how students are motivated. Clarifying and understanding the psychological antecedents of emotions for students is central to improving PE instruction and students' long-term relationship with physical activity (2).

Two influential frameworks converge in understanding and evaluating students' emotions and motivation. First, Self-Determination Theory (SDT) holds that need-supportive teaching – that is, support for autonomy, competence, and relatedness – facilitates autonomous motivation, meaning that students participate in PE out of interest or personal value, which in turn is associated with more positive affective and behavioral outcomes (3). Second, Control-Value Theory (CVT) proposes that students' appraisals of control and value generate discrete emotions during learning; high intrinsic value aligns with enjoyment, while low or externally driven value aligns with boredom or shame. Thus, CVT provides a differentiated account of how cognitive appraisals are linked to specific discrete emotions. Conceptually, CVT's distinction between intrinsic and extrinsic value dovetails with SDT's autonomous and controlled regulation, implying that motivation quality should prospectively relate to changes in discrete emotions during PE (4, 5). Integrating SDT and CVT therefore helps specify a psychological pathway from instructional climate to motivational quality and, ultimately, to students' discrete emotional experiences in PE.

Motivation and emotion are related but distinct. Motivation concerns reasons for action (“Why do I want to do this?”), whereas emotion reflects coordinated affective, cognitive, physiological, motivational, and expressive responses (“How do I feel about this right now?”) (6). Beyond this conceptual distinction, the relevance, quality, and differentiation of both systems develop markedly across school years (7). During adolescence, the salience of self and autonomy intensifies, and aligning activities with self-endorsed values and identity becomes increasingly important (8). These age-graded changes imply that motivational qualities may relate differently to emotional responses in PE across childhood and adolescence. These processes may differ by school level. Elementary students' emotional experiences may depend strongly on immediate situational features such as playfulness, peer interaction, novelty, or teacher support. In adolescence, however, PE increasingly becomes connected to self-evaluation, bodily exposure, social comparison, autonomy, and personally endorsed values. Therefore, autonomous motivation may become a stronger predictor of emotional experiences in secondary PE, because students' feelings are more closely tied to whether PE activities fit their emerging self-concept and values.

Empirically, studies in PE consistently document associations between motivational quality (or value appraisals) and students' emotions (2, 9). However, most evidence is cross-sectional; thus, directionality remains uncertain, and comparisons across school levels are rare. Short-term longitudinal tests embedded in authentic lessons are needed to move beyond simple co-occurrence and toward temporal ordering that is informative for instruction in PE. Existing PE research has often examined positive and negative affect broadly or has treated motivation and emotion as concurrent correlates. Less is known about whether specific motivational qualities prospectively relate to specific discrete emotions. A discrete-emotions perspective therefore allows a more differentiated test of how motivational meaning becomes emotionally expressed in PE.

The present study addresses this gap by examining whether autonomous and controlled motivation predict changes in enjoyment, boredom, anger, and shame across approximately 5-week PE track-and-field units, while accounting for teaching climate. We also explore whether these associations differ between elementary and secondary classes and, for additional support, test the reverse direction (whether emotions predict changes in motivational quality). By aligning SDT and CVT and situating tests within everyday teaching, the study aims to provide actionable evidence for designing lessons that reliably cultivate positive emotional trajectories in PE.

## 2 Methods

### 2.1 Design and setting

This study draws on a short-term longitudinal survey conducted in Germany. Data were collected in both elementary and secondary schools during regular PE lessons using paper-pencil questionnaires. Schools were recruited via existing partnerships with the university's pre-service teacher education program (convenience sampling). A two-wave design was used within regular PE: measures were administered immediately before (T1) and after (T2) approximately 5-week track-and-field units taught across classes.

### 2.2 Participants

In total, *N* = 375 students from 20 classes (8 secondary; 12 elementary) with a mean age of 11.5 years (*SD* = 3.0) participated, 184 identified as female. The secondary-school subsample comprised *n* = 169 students (age: *M* = 14.3 years, *SD* = 2.2); the elementary-school subsample comprised *n* = 206 students (age: *M* = 9.5 years, *SD* = 0.9).

### 2.3 Procedure and ethics

Trained research assistants administered the survey under teacher supervision using standardized instructions. Items were read and explained as needed, and students could ask clarification questions. Questionnaires were completed in class within a single session. Parental consent was obtained prior to data collection. The study received ethical approval from the ethics committee of the first author's university.

### 2.4 Measures

#### 2.4.1 Discrete emotions in PE

Enjoyment, boredom, and anger were assessed as in-activity emotions and shame as an outcome-related emotion using the German Discrete Emotions in Physical Education Scales for secondary and elementary school students (10, 18). Each emotion comprised four items in the secondary version and three items in the elementary version, rated on 5-point Likert scales (1 = strongly disagree, 5 = strongly agree). Internal consistencies (McDonald's *ω*) are reported in Table 1.

**Table 1:** Descriptive statistics by school level.

Variable	Elementary			Secondary			Overall	
	T1	T2	*ω* T1/T2	T1	T2	ω T1/T2	T1	T2
Enjoyment	4.38 ± 0.72 [*n*=206]	3.73 ± 1.07 [*n*=205]	0.90/0.85	3.64 ± 0.93 [*n*=169]	3.78 ± 0.91 [*n*=169]	0.76/0.94	4.05 ± 0.90 [*n*=375]	3.75 ± 1.00 [*n*=374]
Boredom	1.96 ± 0.77 [*n*=206]	1.94 ± 0.80 [*n*=205]	0.69/0.65	2.12 ± 0.92 [*n*=169]	2.00 ± 0.82 [*n*=169]	0.88/0.90	2.03 ± 0.84 [*n*=375]	1.96 ± 0.81 [*n*=374]
Anger	1.72 ± 0.86 [*n*=206]	1.64 ± 0.85 [*n*=205]	0.87/0.87	1.74 ± 0.84 [*n*=169]	1.62 ± 0.72 [*n*=169]	0.93/0.91	1.73 ± 0.85 [*n*=375]	1.63 ± 0.79 [*n*=374]
Shame	1.42 ± 0.61 [*n*=206]	1.88 ± 0.87 [*n*=205]	0.81/0.86	1.79 ± 1.08 [*n*=169]	1.81 ± 1.00 [*n*=169]	0.95/0.95	1.59 ± 0.87 [*n*=375]	1.85 ± 0.93 [*n*=374]
Autonomous motivation	3.05 ± 0.61 [*n*=206]	2.91 ± 0.64 [*n*=204]	0.90/0.92	2.84 ± 0.72 [*n*=169]	2.87 ± 0.71 [*n*=169]	0.94/0.94	2.95 ± 0.67 [*n*=375]	2.89 ± 0.67 [*n*=373]
Controlled motivation	2.10 ± 0.68 [*n*=206]	1.88 ± 0.71 [*n*=204]	0.89/0.93	1.89 ± 0.69 [*n*=169]	1.90 ± 0.66 [*n*=169]	0.92/0.91	2.00 ± 0.69 [*n*=375]	1.89 ± 0.69 [*n*=373]
Need support (overall)	–	2.99 ± 0.51 [*n*=205]	–	–	2.92 ± 0.51 [*n*=169]	–	–	2.96 ± 0.51 [*n*=374]

#### 2.4.2 Motivational regulations

Intrinsic, identified, introjected, and external regulations were measured with a validated German instrument (three items per subscale; prompt: “I participate in PE because …”), using 4-point scales (1 = strongly disagree, 4 = strongly agree) (11). Following Self-Determination Theory, we computed composite indices of autonomous motivation (intrinsic+identified) and controlled motivation (introjected+external).

#### 2.4.3 Perceived need-supportive teaching

Students reported perceived autonomy, competence, and relatedness support in PE (4-point scales) after the unit (e.g., “My PE teacher often provides me with choices and options”), and were instructed to respond with the just-completed unit in mind. To capture need-supportive teaching, we aggregated these three subscales and then computed the class-mean across students within each class (3). This class-level operationalization reflects the assumption that students within the same class were exposed to a common teacher, content structure, and set of instructional conditions during the track-and-field unit. Empirical support for aggregation was indicated by substantial between-class variability and reliable class means, as reported below.

### 2.5 Data analysis

We screened the data for out-of-range values and implausible response patterns; no cases were excluded. Item-level missingness was very low (≤ 0.5%). Scale scores were computed as available-item means. The class-mean need support index (T2) was entered as a class-level covariate.

Primary analyses used linear mixed-effects models with a random intercept for class to account for nesting. For each emotion, T2 scores were regressed on the corresponding T1 emotion (autoregressive control), autonomous and controlled motivation (T1), school level, gender, and the class-mean need support covariate. Because the corresponding baseline emotion was included, coefficients for motivational regulation can be interpreted as associations with baseline-adjusted change in emotions across the unit rather than as cross-sectional associations with T2 emotion levels (12). The class random intercept accounts for similarities among students from the same class, and the class-level need-supportive teaching variable captures shared differences in the perceived instructional climate across the track-and-field unit. Thus, the models estimate whether individual motivational quality predicts emotional change over and above prior emotion levels and between-class differences in teaching climate (13).

To probe directionality, we estimated parallel reverse models in which motivation at T2 was regressed on its T1 baseline and on T1 emotions. Each model used the same controls and structure as the primary analyses. Coefficients for T1 emotions in these models index prospective change in motivation from T1 to T2 conditional on baseline. Interaction terms were examined when initial models indicated relevant main effects. Models with and without interactions were compared using ΔAIC, ΔBIC and likelihood-ratio tests. Assumptions were checked via variance inflation factors (VIF) for multicollinearity and tests of residual heteroscedasticity. Analyses were conducted in RStudio.

## 3 Results

### 3.1 Descriptive statistics and reliability

Across both school levels, enjoyment was high at baseline and, on average, slightly lower at T2 in the pooled sample; boredom and anger were low and remained low; shame was lower at T1 and higher at T2 (Table 1). Internal consistencies were acceptable to excellent for emotions and motivation (McDonald's *ω* = 0.69–.95), with lower reliability for elementary-school boredom at T2 (*ω* = 0.65). Autonomous and controlled motivation showed high reliability in both samples (*ω* = 0.89–.94). Internal consistencies were acceptable to good for the need support subscales (autonomy/competence/relatedness; *ω* = 0.65–.80). Students' need support ratings showed substantial between-class variability [ICC(1) = 0.20] and high reliability of class-means [ICC(2) = 0.82]. We therefore aggregated autonomy, competence, and relatedness support to a class-mean need support index used as a class-level covariate.

### 3.2 Assumption and model checks

Across models, multicollinearity was low (all VIFs = 1.0–1.8), and homoscedasticity tests were non-significant (ps ≥ 0.14). Intraclass correlations indicated meaningful clustering for enjoyment (ICC = 0.34) and shame (ICC = 0.30), and small clustering for boredom (ICC = 0.09) and anger (ICC = 0.04). Conditional *R*^2^ values ranged from 0.28 to 0.60; marginal *R*^2^ from 0.24 to 0.43 (Tables 2, 3).

**Table 2:** Fixed effects from linear mixed-effects models predicting T2 enjoyment and boredom.

Predictor	Enjoyment Model 1			Enjoyment Model 2			Boredom		
	*b* (*SE*)	95% CI	*p*	*b* (*SE*)	95% CI	*p*	*b* (*SE*)	95% CI	*p*
(Intercept)	−2.94 (1.49)	[−5.88, −0.01]	0.049	−2.38 (1.49)	[−5.30, 0.55]	0.111	3.44 (0.81)	[1.85, 5.02]	< 0.001
Enjoyment T1	0.41 (0.06)	[0.30, 0.53]	< 0.001	0.41 (0.06)	[0.29, 0.52]	< 0.001			
Boredom T1							0.35 (0.05)	[0.25, 0.45]	< 0.001
Auton. Motiv.	0.21 (0.07)	[0.07, 0.35]	0.003	0.03 (0.10)	[−0.17, 0.23]	0.786	−0.13 (0.06)	[−0.25, −0.01]	0.035
Contr. Motiv.	0.03 (0.05)	[−0.07, 0.14]	0.552	0.05 (0.05)	[−0.06, 0.15]	0.385	0.07 (0.05)	[−0.03, 0.18]	0.172
Secondary (vs. Elementary)	0.54 (0.24)	[0.06, 1.01]	0.028	−0.38 (0.40)	[−1.16, 0.41]	0.349	−0.09 (0.13)	[−0.34, 0.16]	0.494
Female (vs. Male)	−0.16 (0.07)	[−0.30, −0.02]	0.023	−0.24 (0.32)	[−0.87, 0.39]	0.460	−0.04 (0.07)	[−0.18, 0.09]	0.529
Need Support	1.43 (0.49)	[0.47, 2.40]	0.004	1.42 (0.48)	[0.47, 2.37]	0.004	−0.64 (0.26)	[−1.14, −0.14]	0.013
Auton. Motiv. x Secondary (vs. Elementary)				0.31 (0.11)	[0.10, 0.53]	0.005			
Auton. Motiv. x Female (vs. Male)				0.03 (0.11)	[−0.18, 0.24]	0.775			
*SD* (Intercept)	0.48			0.47			0.21		
*SD* (Observations)	0.66			0.66			0.67		
Marginal/Conditional *R^2^*	.36/.58			.38/.59			.24/.31		

b, unstandardized regression coefficient; SE, standard error; CI, confidence interval. SD (Intercept) refers to the estimated standard deviation of the class-level random intercept; SD (Observations) refers to the residual standard deviation at the student level.

**Table 3:** Fixed effects from linear mixed-effects models predicting T2 shame and anger.

Predictor	Shame			Anger		
	*b* (*SE*)	95% CI	*p*	*b* (*SE*)	95% CI	*p*
(Intercept)	4.60 (1.27)	[2.10, 7.10]	< 0.001	1.36 (0.63)	[0.11, 2.60]	0.033
Shame T1	0.60 (0.04)	[0.51, 0.69]	< 0.001			
Anger T1				0.44 (0.04)	[0.35, 0.52]	< 0.001
Auton. Motiv.	0.05 (0.05)	[−0.05, 0.15]	0.353	0.06 (0.05)	[−0.05, 0.17]	0.282
Contr. Motiv.	0.02 (0.05)	[−0.08, 0.12]	0.716	0.07 (0.05)	[−0.03, 0.17]	0.164
Secondary (vs. Elementary)	−0.40 (0.21)	[−0.81, 0.00]	0.051	−0.04 (0.10)	[−0.24, 0.15]	0.656
Female (vs. Male)	0.06 (0.07)	[−0.07, 0.19]	0.389	0.00 (0.07)	[−0.14, 0.14]	0.964
Need Support	−1.27 (0.42)	[−2.10, −0.45]	0.002	−0.27 (0.20)	[−0.66, 0.13]	0.183
*SD* (Intercept)	0.40			0.14		
*SD* (Observations)	0.61			0.67		
Marginal/Conditional *R*^2^	.43/.60			.25/.28		

b, unstandardized regression coefficient; SE, standard error; CI, confidence interval. SD (Intercept) refers to the estimated standard deviation of the class-level random intercept; SD (Observations) refers to the residual standard deviation at the student level.

### 3.3 Main models

In the baseline model for enjoyment (Model 1), higher T1 enjoyment predicted higher T2 enjoyment (*b* = 0.41, *p* < 0.001). Autonomous motivation at T1 predicted T2 enjoyment (*b* = 0.21, *p* = 0.003), and class-mean need support was also positive (*b* = 1.43, *p* = 0.004). Secondary students showed higher baseline-adjusted enjoyment than elementary students after the unit (*b* = 0.54, *p* = 0.028). Girls showed a slightly lower baseline-adjusted enjoyment than boys (*b* = −0.16, *p* = 0.023); controlled motivation was not uniquely related (Table 2).

Adding interaction terms (Model 2) improved fit (*Δ*AIC = −4.05, *Δ*BIC = −3.80 LR = 8.05, *df* = 2, *p* = 0.018). The autonomous-motivation×school-level interaction was significant (*b* = 0.31, *p* = 0.005), indicating that autonomous motivation was more strongly associated with baseline-adjusted enjoyment in secondary classes. Simple-slope analyses showed no clear association in elementary classes (b = 0.04), but a positive association in secondary classes [b = 0.35, 95% CI (0.18, 0.53)]. Thus, students' autonomous reasons for participating in PE were particularly predictive of enjoyment trajectories in secondary PE.

Higher T1 boredom predicted higher T2 boredom (*b* = 0.35, *p* < 0.001). Autonomous motivation was associated with a decrease in boredom (*b* = −0.13, *p* = 0.035), and class-mean need support was also negative (*b* = −0.64, *p* = 0.013). Controlled motivation as well as school level and gender were not uniquely associated (Table 2). For shame, T1 shame robustly predicted T2 shame (*b* = 0.60, *p* < 0.001), and class-mean need support related to lower shame (*b* = −1.27, *p* = 0.002). The secondary vs. elementary contrast did not reach conventional statistical significance (*p* = 0.051); autonomous and controlled motivation were not uniquely associated (Table 3). For anger, T1 anger predicted T2 anger (*b* = 0.44, *p* < 0.001); other predictors were not unique correlates (Table 3).

We tested whether T1 emotions predicted changes in motivation from T1 to T2 (controlling for baseline motivation, school level, gender, and need support). T1 enjoyment did not predict autonomous motivation at T2 (*b* = 0.04, *p* = 0.40), whereas baseline autonomous motivation was a strong positive predictor (*b* = 0.55, *p* < 0.001). Class-mean need support was positively associated with autonomous motivation (*b* = 0.52, *p* = 0.03). For controlled motivation, T1 boredom did not predict T2 controlled motivation (*b* = 0.05, *p* = 0.14), while baseline-controlled motivation was strongly predictive (*b* = 0.48, *p* < 0.001). Need support showed a negative association with controlled motivation (*b* = −0.42, *p* = 0.06).

## 4 Discussion

Viewed through SDT and CVT together, the purpose of this study was to examine whether students' motivational quality is linked to short-term changes in discrete emotions during authentic PE units. The findings support a differentiated motivational–emotional pattern. Autonomous motivation predicted more favorable emotional trajectories, namely higher baseline-adjusted enjoyment and lower baseline-adjusted boredom. Controlled motivation, in contrast, showed no unique associations once autonomous motivation, prior emotion levels, school level, gender, and teaching climate were considered. At the class level, need-supportive teaching was associated with higher enjoyment and lower boredom and shame. Together, these findings suggest that both individual motivational quality *and* shared motivational climate contribute to the emotional experiences during PE lessons (3).

Autonomous reasons to participate in PE were linked to enjoyment particularly in secondary classes. This finding is consistent with developmental perspectives suggesting that adolescence is marked by a growing concern with autonomy, identity, self-evaluation, and personally endorsed values (7, 8). From an SDT perspective, identity development involves the exploration and internalization of personally meaningful values, goals, and behaviors (14). In secondary PE, students may therefore evaluate activities more strongly in relation to whether they fit their interests and emerging sense of self (15). Consequently, the question of “why I participate” may become more closely connected to “how I feel while participating.” In elementary classes, by contrast, enjoyment may be more strongly shaped by immediate situational qualities of the lesson. This interpretation is consistent with research on primary PE showing that children's enjoyment is closely connected to concrete teaching practices and lesson experiences, such as variety, challenge, and social interaction (16). This may explain why autonomous motivation was a clearer predictor of enjoyment trajectories in secondary than in elementary PE.

When autonomous motivation and climate were considered, the null findings for controlled regulation aligned with prior evidence that external reasons are comparatively weak predictors of affective outcomes when motivation is disaggregated (3). Reverse analyses further support the directional emphasis of the present study: emotions did not predict changes in motivation across the unit. This pattern may reflect both the comparatively greater stability of motivational regulations over a short five-week period and the fact that the track-and-field units were regular PE units rather than interventions specifically designed to change students’ motivational quality. Discrete emotions, by contrast, are more proximal responses to situational appraisals and may therefore be more sensitive to short-term instructional experiences. Practically, this asymmetry suggests that strengthening autonomous reasons for participation and establishing need-supportive instructional conditions may be a more reliable route to improving students' in-activity emotions than assuming that short-lived positive emotional experiences will, by themselves, translate into higher-quality motivation (5).

Need-supportive teaching was uniquely related to lower shame. This finding is theoretically plausible because shame is an outcome-related emotion that is closely tied to perceived failure, social evaluation, and threats to the self. In PE, such experiences may be especially salient because performance is often public and bodily visible. A climate that supports competence, relatedness, and autonomy may reduce the likelihood that students interpret difficulties as personally devaluing or socially exposing. From a pedagogical perspective, this is important because shame may not always appear as overt disengagement; students can comply with tasks while nevertheless experiencing PE as threatening or humiliating. Teachers should therefore attend not only to visible participation, but also to whether tasks, feedback, and comparison structures protect students' sense of competence and belonging.

Anger showed a different pattern. Apart from baseline anger, none of the motivational or contextual predictors showed unique associations with T2 anger. This may indicate that anger in PE is less strongly tied to general motivational quality and more dependent on immediate events, such as perceived unfairness, peer conflict, rule disputes, or blocked goals (4).

Beyond prior cross-sectional studies [e.g., (2)], our short-term longitudinal models provide prospective evidence by adjusting for baseline emotions, accounting for class clustering and climate, and testing reverse paths. Such prospective evidence is still limited in PE motivation-emotion research. While still observational, these features move the evidence closer to causal inference regarding motivation-emotion change across PE lessons. The focus on a single content area, track-and-field, enhances internal validity but limits generalizability to other PE contents. At the same time, track-and-field provides an informative context for studying discrete emotions such as boredom and shame because tasks can be repetitive, individual performance is often visible, and outcomes are comparatively easy to observe and compare. In other PE contents, the motivational and emotional affordances may differ. In team games or cooperative tasks, emotions may depend more strongly on peer interaction, tactical involvement, group belonging, and shared task goals. Dance and gymnastics may also involve high bodily exposure and public performance, but they differ from track-and-field in the nature of performance demands, the role of expressiveness or movement quality, and the gendered meanings often attached to these activities (17). Future studies should therefore examine whether the motivational–emotional patterns observed here replicate across PE contents that differ in visibility, evaluative structure, cooperation, expressiveness, and gendered expectations.

Several limitations should be considered. First, the study was observational; although the autoregressive design strengthens temporal interpretation, the findings should not be read as causal effects. Second, the two-wave design allows baseline-adjusted prediction but does not capture reciprocal motivational–emotional dynamics across individual lessons. Third, boredom showed lower reliability in the elementary-school sample, which may reflect younger students' difficulty in differentiating boredom from related low-arousal states or from a more general dislike of the activity (18). Findings involving boredom in the elementary subsample should therefore be interpreted with some caution. Finally, need-supportive teaching was modeled as a class-level indicator of shared instructional climate. Although this was consistent with the study's focus and supported by the aggregation indices, this approach did not capture within-class differences in students' individual perceptions of teacher support.

## Data Availability

The datasets presented in this article are not readily available because the datasets generated and analyzed during the current study are not publicly available and cannot be shared because the informed consent obtained from participants and their legal guardians did not include permission for data sharing. Requests to access the datasets should be directed to michael.braksiek@uni-vechta.de.
